# Risk of musculoskeletal disorder among Taiwanese nurses cohort: a nationwide population-based study

**DOI:** 10.1186/1471-2474-14-144

**Published:** 2013-04-23

**Authors:** Yueh-Chin Chung, Chin-Tun Hung, Shu-Fen Li, Horng-Mo Lee, Shyang-Guang Wang, Shu-Chuan Chang, Lee-Wen Pai, Chien-Ning Huang, Jen-Hung Yang

**Affiliations:** 1 Institute of Medicine, Chung Shan Medical University, Taichung, Taiwan; 2 Department of Nursing, Central Taiwan University of Science and Technology, Taichung, Taiwan; 3 Department of Healthcare Administration, Central Taiwan University of Science and Technology, Taichung, Taiwan; 4 Institute of Pharmaceutical Science and Technology; Department of Medical Laboratory Science and Biotechnology, Central Taiwan University of Science and Technology, Taichung, Taiwan; 5 Department of Internal Medicine, Chung Shan Medical University Hospital, Taichung, Taiwan; 6 College of Medicine, Tzu Chi University; Department of Dermatology, Buddhist Tzu Chi General Hospital, Taiwan, No.701, Zhongyang Rd., Sec.3, Hualien, 97004, Taiwan

**Keywords:** Musculoskeletal disorders, Nurses, Low back pain, Epidemiology, Incidence

## Abstract

**Background:**

Musculoskeletal disorders (MSDs) represent the leading causes of occupational injuries among nursing staff. This population-based study was designed to assess the incidence and age-specific incidence of MSDs among a Taiwanese nurse cohort compared with non-nurses.

**Description:**

Data from the Taiwan National Health Insurance Research Database were used to identify MSDs in the study population. A total of 3914 nurses with a diagnosis of MSD were included, together with 11,744 non-nurses as a comparison group. The comparison subjects were randomly selected at a ratio of 3:1 relative to the nurse population and were matched by gender and age. The incidence of MSDs was calculated for the study group, with nurse-to-reference risk ratios presented as odds ratios with 95% confidence intervals (CIs). During the period 2004–2010, 3004 MSDs occurred among the nurses (76.24%) and 7779 (65.79%) in the non-nurses. The annual incidence of MSDs for the nurses increased from 28.35% in 2006 to 33.65% in 2010. The nurse-to-reference risk ratio was 1.27 (95% CI 1.19–1.35) in 2004 and 1.46 (1.37–1.55) in 2010. Herniation of intervertebral disc, lumbago, rotator cuff syndrome, medial epicondylitis, trigger finger and carpal tunnel syndrome were the most common problems.

**Conclusions:**

Nurses are at higher risk of MSDs and the trend is increasing. Incorrect work-related posture/movement, psychological issues and the rolling shift system may be the major causes of MSDs among nurses in Taiwan.

## Background

Musculoskeletal disorders (MSDs) are inflammatory and degenerative conditions that affect the muscles, tendons, ligaments, joints or peripheral nerves, usually leading to ache, pain or discomfort [1,2]. MSDs are generally caused by repetitive manual labor, lifting heavy loads, overexertion, or working in an awkward posture. MSDs can have a serious impact on quality of life and may result in work restriction, absenteeism or even the need to change jobs [3-5].

The incidence of MSDs among healthcare workers is high, particularly in those who are involved in patient handling [6-8]. Nurses routinely perform activities including lifting and moving patients, which are known risk factors for MSDs [2,9-11]. Low back pain (LBP) is the most common MSD in the nursing profession [12,13], accounting for 35–80% of the lifetime incidence [14-16].

MSDs seem to be prevalent among nurses, but observations in the literature based on questionnaires as measurement tools may not reflect the true situation throughout Taiwan. According to this report questionnaire is often influenced by information bias that should be minimized [17]. Clinical data on MSDs among nurses may better represent the importance of this issue, to obtain which a population-based longitudinal survey is required.

Taiwan’s National Health Insurance Research Database (NHIRD) derives from the Taiwan National Health Insurance (NHI) program and is maintained by the Taiwan National Health Research Institute (NHRI), which updates data annually from the NHI program and classifies them for the NHIRD. In 2008, 22.89 million of Taiwan’s 22.96 million people (99.7% of the island’s population) were covered by the NHI program [18], a mandatory system that every person is required to join. In 2008, about 20.93 million persons claimed insurance, representing 91% of the insured population. More than 300 studies based on the NHIRD have been published in peer-reviewed journals. Using the NHIRD, the present study sought to determine the incidence and risk of MSDs in Taiwanese nurses and the particular MSDs for which they are most at risk.

## Construction and content

### Methods

The Taiwanese National Health Insurance Bureau (NHIB) provides electronic data including patients’ sex and birth date, the classification codes of diseases diagnosed, health services received, and the clinic or hospital code. The NHIB collects data from the NHI program and sorts it into data files each year, including registration files and original claim data for reimbursement. These data files are de-identified by scrambling the identification codes of both patients and medical facilities and are sent to the NHRI to form the NHIRD. The data used in this study were retrieved from a representative NHIRD cohort from January 2004 to December 2010. One million subjects were used for this longitudinal study. The sample size of 3914 nurses was selected randomly from the approximately 90,022 registered nurses in the NHI database. The nurses included in this study comprised 3861 women and 53 men. The reference population of 11,744 subjects was selected from 270,802 individuals whose occupation was not nursing and included both working and non-working subjects. Access to the NHIRD was approved by the NHRI Review Committee.

Cases of MSD were identified from the NHIRD for the years 2004 to 2010 using the 9th revision of the International Classification of Diseases, Clinical Modification (ICD-9-CM), for both the registered nurses and the reference population. Abridged codes (A-codes) were converted into ICD-9-CM codes for data analysis. We excluded tumors (ICD-9-CM codes 170, 171 and 213) and trauma (injury or poisoning, ICD-9-CM codes 800–999). A MSD was defined as a diagnosis of either ICD-9-CM code 710–739 or A-code 431–439. These MSDs include rotator cuff syndrome of the shoulder (ICD-9-CM 726.1), medial epicondylitis (ICD-9-CM 726.31), lateral epicondylitis (ICD-9-CM 726.32), trigger finger (ICD-9-CM 727.03), radial styloid tenosynovitis (ICD-9-CM 727.04) and carpal tunnel syndrome (ICD-9-CM 354.0). Prolonged standing and lifting of heavy objects may contribute to LBP, so the incidences of nursing-related LBP, including lumbar spondylosis (ICD-9-CM 721.3), herniated intervertebral disc (HIVD) (ICD-9-CM 722.10), lumbago (ICD-9-CM 724.2) and backache (ICD-9-CM 724.5) were also compared between the two groups [19].

We first calculated the overall incidence of MSDs in the two study groups for the years 2004–2010. The annual incidence was the number of new cases of MSD divided by the size of the population at risk in each year. For example, the 2004 incidence was 1145/3914, where 1145 is the number of new MSD cases in 2004 and 3914 was obtained by subtracting the number of MSD cases in 2003 from the population at risk in 2004. The seven-year cumulative incidence was the number of new cases of MSDs divided by the size of the population at risk from 2004 to 2010. The annual incidence of MSDs was calculated for the study group, with the nurse-to-reference risk ratio presented as the odds ratio (OR) with its 95% confidence interval (CI) [20]. The age-specific and site-specific incidences of MSDs during that period were also compared between the two groups.

### Results

A total of 3914 nurses and three times this number of reference subjects (11,744) were selected from the reimbursement claims database of the NHI of Taiwan. The gender distribution of the nurses (98.64% women and 1.36% men) was comparable to that of the references, as was the age distribution. The seven-year cumulative incidence of MSDs was higher among the nurses (76.24%) than in the reference group (65.79%) (Table 1).

**Table 1:** Demographic data and seven-year cumulative incidence of musculoskeletal disorders (MSDs) in nurses and reference group

		**Nurses**	**Reference group**
*N*		3914	11,744
Gender	Female	3861 (98.64%)	11,585 (98.64%)
	Male	53 (1.36%)	159 (1.36%)
Age (years)	Female	34.02 ± 7.72	34.05 ± 7.73
	Male	31.15 ± 4.74	31.15 ± 4.71
**Seven-year cumulative incidence of MSDs**^**a**^		3004 (76.24%)	7779 (65.79%)

^a^Seven-year cumulative incidence is the number of new cases of MSD divided by the size of the population at risk from 2004 to 2010.

The annual incidences of MSDs during the period 2004–2010 were significantly higher among the nurses than the references (all *p* < 0.001), with nurse-to-reference risk ratios ranging from 1.27 to 1.46 (Table 2). Notably, there was a trend of increasing incidence from 2005 (28.45%) to 2010 (33.65%). These data suggest that the nurses were more susceptible to MSDs than the non-nurse references, and that the annual incidence increased over a five-year period.

**Table 2:** Annual incidence of musculoskeletal disorders (MSDs) in nurses and reference group, 2004–2010

**Year**	**Annual incidence**^**a **^**(%)**				
	**Nurses**	**Reference group**	**Odds ratio**	**95% Confidence interval**	***p***
**2004**	29.26	24.58	1.27	1.19–1.35	< 0.001
**2005**	28.45	23.84	1.27	1.19–1.35	< 0.001
**2006**	28.35	23.32	1.30	1.22–1.39	< 0.001
**2007**	30.20	24.37	1.34	1.26–1.43	< 0.001
**2008**	29.89	24.37	1.32	1.24–1.41	< 0.001
**2009**	33.53	26.46	1.40	1.32–1.49	< 0.001
**2010**	33.65	25.80	1.46	1.37–1.55	< 0.001

^a^Annual incidence is the number of new cases of MSD divided by the size of the population at risk in each year.

The age-specific incidence of MSDs ranged from 56.00% to 85.61% among the nurses. In both nurses and references, the age group with the highest incidence was the 20–24-year-olds and that with lowest was the ≥ 60 year-old group. The age-specific nurse-to-reference risk ratios for the annual incidences of MSDs for 2004–2010 ranged from 0.92 to 2.29. In all age groups, the age-specific incidence of MSDs was consistently higher in the nurses than in the reference group (all *p* < 0.01) (Table 3).

**Table 3:** **Average age-specific incidence**^**a **^**of musculoskeletal disorders (MSDs) among nurses and reference group, 2004–2010**

**Age (years)**	**Nurses**	**Reference group**	**Odds ratio**	**95% Confidence interval**	***p***
**20–24**	85.61	72.20	2.29	2.13–2.46	< 0.001
**25–29**	73.60	65.78	1.45	1.36–1.54	< 0.001
**30–34**	61.26	52.61	1.42	1.35–0.51	< 0.001
**35–39**	77.83	61.54	2.19	2.06–2.33	< 0.001
**40–44**	67.99	61.00	1.36	1.28–1.44	< 0.001
**45–49**	75.24	66.56	1.53	1.44–1.62	< 0.001
**50–54**	66.45	68.36	0.92	0.86–0.97	< 0.01
**55–59**	56.12	36.43	2.23	2.11–2.36	< 0.001
**≥ 60**	56.00	46.67	1.45	1.38–1.54	< 0.001

^a^Incidence is the number of new cases of MSD divided by the size of the population at risk from 2004 to 2010.

The incidences of MSDs in different parts of the body were investigated for the year 2010. Table 4 shows that the incidences of rotator cuff syndrome, medial and lateral epicondylitis, trigger finger, carpal tunnel syndrome, lumbar spondylosis, HIVD, lumbago and backache were significantly higher in the nurses than in the reference group (all *p* < 0.05).

**Table 4:** Incidence and odds ratio (OR) for specific sites of musculoskeletal disorders (MSDs) in nurses and reference group in 2010

**ICD-9-CM code**^**a**^		**Incidence**^**b **^**(%)**			
		**Nurses**	**Reference group**	**OR (95% Confidence interval)**	***p***
**726.1**	Rotator cuff syndrome	0.83	0.53	4.33 (2.51–7.47)	< 0.001
**726.31**	Medial epicondylitis	0.25	0.09	3.37 (1.49–7.63)	< 0.01
**726.32**	Lateral epicondylitis	0.58	0.39	2.16 (1.23–3.79)	< 0.01
**727.03**	Trigger finger	0.48	0.26	2.63 (1.45–4.75)	< 0.01
**727.04**	Radial styloid tenosynovitis	0.30	0.20	1.71 (0.90–3.27)	0.102
**354.0**	Carpal tunnel syndrome	0.79	0.63	2.21 (1.18–4.13)	< 0.05
**721.3**	Lumbar spondylosis	1.07	0.81	1.36 (1.00–1.84)	< 0.05
**722.10**	Herniated intervertebral disc	1.45	0.64	2.48 (1.82–3.38)	< 0.001
**724.2**	Lumbago	7.08	5.43	2.04 (1.70–2.45)	< 0.001
**724.5**	Backache	4.34	3.14	1.68 (1.40–2.01)	< 0.001

^a^ICD-9-CM, 9th revision of the International Classification of Diseases, Clinical Modification.

^b^Annual incidence is the number of new cases of MSD divided by the size of the population at risk in each year.

We next examined the age-specific incidence of LBP from 2004 to 2010; this was consistently higher among the nurses than the references (*p* < 0.01), with the exception of the 55–59-year-old group (Table 5). The nurse-to-reference OR was highest in 20–24-year-olds (OR, 1.60; 95% CI, 2.67–2.97) and those aged ≥ 60 years (OR, 2.14; 95% CI, 2.02–2.26). These data suggest that the oldest populations were the most vulnerable.

**Table 5:** Average age-specific incidence of low back pain (LBP) among nurses and reference group in 2004–2010

**Age (years)**	**Incidence**^**a **^**(%)**		**Odds ratio**	**95% Confidence interval**	***p***
	**Nurses**	**Reference group**			
**20–24**	60.35	48.79	1.60	2.67–2.97	< 0.001
**25–29**	56.09	48.34	1.36	1.29–1.44	< 0.001
**30–34**	52.30	41.83	1.52	1.44–1.61	< 0.001
**35–39**	57.52	45.03	1.65	1.56–1.75	< 0.001
**40–44**	57.17	49.00	1.39	1.31–1.47	< 0.001
**45–49**	59.85	57.76	1.09	1.03–1.15	< 0.01
**50–54**	60.08	58.43	1.07	1.01–1.13	< 0.05
**55–59**	50.21	49.23	1.04	0.98–1.10	0.166
**≥ 60**	56.00	37.33	2.14	2.02–2.26	< 0.001

^a^Incidence is the number of new cases of LBP divided by the size of the population at risk from 2004 to 2010.

## Utility

Musculoskeletal disorders (MSDs) represent the leading causes of occupational injuries among nurses. Our findings nurses are at higher risk of MSDs and the trend is increasing that considered when developing policies for the prevention of MSDs in the workplace, particularly with respect to occupation-associated injuries. Education program on prevention for MSDs and rolling shift system strategies may be helpful in reducing the incidence of MSDs.

## Discussion

A high incidence of MSDs among nurses has been demonstrated in numerous reports [21-24]. However, most of these surveys used questionnaires as measurement tools, which may not be compatible with the clinical diagnosis and evaluation of MSDs. The strength of this study is that most of the nurses in Taiwan were included for statistical analysis. The NHI in Taiwan is a mandatory universal health insurance program with more than 96% coverage [18]. Because of the use of randomly selected reference subjects, the measurement of MSD incidence in the present study was reliable and similar to that obtained using the whole population as the denominator. Thus, information bias was substantially reduced. To our knowledge, no previous study has used nationwide health insurance data to investigate MSDs in nurses. However, this study is limited with respect to its generalizability because the incidence of MSDs was work related. In addition, not all nurses suffering MSDs seek medical attention that would be listed in the claim files. The database used in this study cannot provide information on an occupation-matched reference population; thus, another limitation of the present study is that we were unable to include both working and non-working populations as reference subjects. We can also assume that not every nurse with a MSD receives the same treatment as the non-nurse general public. Our statistical analysis was designed simply to indicate whether nurses have a higher incidence of MSDs than the general public.

This study demonstrated a higher incidence of MSDs among nurses. Our results agree with those published by Tinubu et al. [25], who reported a lifetime MSD incidence of 84.4% among nurses. Similar incidences of MSDs in nurses have been reported by other studies of various populations [6,24]. The seven-year cumulative incidence in Taiwan was 76.24%, which is consistent with those previous reports. However, the incidence of MSDs among nurses was higher than that found in Chinese restaurant cooks, hotel-servers and community food service workers in Taiwan [26,27]. The relatively high incidence among our nurse cohort suggests that nurses work in a relatively high-risk environment for MSDs. The peak of age-specific MSD incidence in 20–24-year-olds (85.61%) warrants further discussion. Musculoskeletal pain has become a major complaint among young nurses and is increasingly occurring at a younger age [28,29]. The average age and seniority of nursing personnel are 26 and 4.73 years, respectively [30], and our findings should be considered when developing policies for the prevention of MSDs in the workplace, particularly with respect to occupation-associated injuries.

Lumbago, backache and HIVD were the most common site-specific disorders. LBP is prevalent among nurses, nursing aides and nursing students [31]. The seven-year cumulative incidence of LBP was significantly higher in the nurse cohort (58.76%) than in the reference group (49.02%). This large difference may indicate which is the most vulnerable part of the body in nurses at work. The high incidence of lumbago may be a consequence of nurses spending long periods of time standing, and their lifting and moving of patients.

Chiou et al. [32] reported that the lifetime incidence of LBP is 77.9%. Nursing tasks involving heavy physical labor and standing with the trunk in a bent or twisted position were the most common sources of back pain among nurses [7,9,33]. Overexertion of the back muscles can injure or tear ligaments in the back, which in turn leads to pain. Coping strategies include change of working technique, use of lifting equipment and avoiding strenuous tasks at work [15,34-36]. Educational programs on the prevention of or coping strategies for MSDs may be helpful in reducing their incidence [5,37].

In a questionnaire survey, Chen and Ting found that MSDs in nurses in Taiwan are primarily due to high workload and work pressure [38,39]. In other studies, Feng et al. and Nien found that 94.3% of Taiwanese nurses thought that MSDs may be caused by incorrect work-related posture/movement, insufficient rest or psychological conditions. They also considered the rolling shift system as a major cause of MSDs among Taiwanese nurses [40,41].

## Conclusions

The present study demonstrates that Taiwanese nurses are at higher risk of MSDs than the non-nurse population. The most critical cause for such MSDs was lack of training to be aware of the associated work-related situations. Education program may be helpful in reducing the incidence of MSDs.

## Availability and requirements

In summary, the present study demonstrates that MSDs may represent a significant burden for nurses. The annual incidence of MSDs for the nurses was increased in the years 2006 to 2010. The age-specific incidences of MSDs of nurse were highest in the populations of 20–24-year-old and those aged over 60. The Lumbago and back are the body parts most affected by MSDs, suggesting that education of ergonomics should be included in the nurse curriculum to reduce risks of MSDs in the future.

## Abbreviations

MSD: Musculoskeletal Disorder; LBP: Low Back Pain; NHIRD: National Health Insurance Research Database; NHI: National Health Insurance; NHRI: National Health Research Institute; NHIB: National Health Insurance Bureau; ICD-9-CM: 9th revision of the International Classification of Diseases, Clinical Modification; HIVD: Herniated Intervertebral Disc; OR: Odds Ratio; CI: Confidence Interval.

## Competing interests

The authors declare that they have no competing interests.

## Authors’ contributions

YCC, CTH, SFL and HML participated in the conception and design of the study. YCC, SCC, HML and CNH performed the literature search and selection of studies. SFL, SGW, SCC, LWP and JHY extracted relevant data. CTH, HML and JHY participated in the analysis and interpretation of data and in the preparation and revision of the manuscript. All authors read and approved the final manuscript.

## Pre-publication history

The pre-publication history for this paper can be accessed here:

http://www.biomedcentral.com/1471-2474/14/144/prepub
